# Arithmetic in two languages: Localizing simple multiplication processing in the adult bilingual brain

**DOI:** 10.1162/imag_a_00199

**Published:** 2024-06-24

**Authors:** Vanessa R. Cerda, Macarena Suárez-Pellicioni, James R. Booth, Nicole Y. Wicha

**Affiliations:** Department of Psychology and Human Development, Vanderbilt University, Nashville, TN, United States; Department of Educational Studies in Psychology, Research Methodology, and Counseling, University of Alabama, Tuscaloosa, AL, United States; Department of Neuroscience, Developmental and Regenerative Biology, University of Texas at San Antonio, San Antonio, TX, United States

**Keywords:** bilingualism, arithmetic, neuroimaging, problem size effect, multiplication, numerical cognition

## Abstract

Verbally memorized multiplication tables are thought to create language-specific memories. Supporting this idea, bilinguals are typically faster and more accurate in the language in which they learned math (LA+) than in their other language (LA-). No study has yet revealed the underlying neurocognitive mechanisms explaining this effect, or the role of problem size in explaining the recruitment of different brain regions in LA+ and LA-. To fill this gap in the literature, 29 Spanish-English early bilingual adults, proficient in both languages, verified simple multiplication problems in each language while functional magnetic resonance imaging (fMRI) was acquired. More specifically, this study aimed to answer two questions: 1) Does LA+ recruit left superior and middle temporal gyri (STG/MTG) to a greater extent than LA-, reflecting more robust verbal representations of multiplication facts in LA+? In contrast, does LA- recruit the inferior frontal gyrus (IFG), reflecting more effortful retrieval, or the intraparietal sulcus (IPS), reflecting reliance on quantity processes? 2) Is there an interaction between language and problem size, where language differences are more pronounced for less practiced, large multiplication problems (e.g., 8 x 9) in comparison to more familiar, small problems (e.g., 2 x 3). Functional localizer tasks were used to identify hypothesis-driven regions of interest in verbal areas associated with verbal representations of arithmetic facts (left STG/MTG) and with the effortful retrieval of these facts (left IFG) and quantity areas engaged when calculation-based strategies are used (bilateral IPS). In planned analyses, no cluster reached significance for the direct comparison of languages (question 1) or for the interaction between language and problem size (question 2). An exploratory analysis found a main effect of problem size, where small problems recruited left STG/MTG and left IFG to a greater extent than large problems, suggesting greater verbal involvement for these problems in both languages. Additionally, large problems recruited right IPS to a greater extent than small problems, suggesting reliance on quantity processes. Our results suggest that proficient early bilingual adults engage similar brain regions in both languages, even for more difficult, large problems.

## 1 Introduction

Simple arithmetic facts, like multiplication tables, are thought to engage language-specific memories because bilinguals typically learn and prefer to perform simple arithmetic in the language they learned the facts inDewaele (2007)andVaid and Menon (2000). Bilinguals often show faster and more accurate responses in the language in which they learned math (LA+) than their other language (LA-) (Cerda et al., 2019,^2023^;Dehaene et al., 1999;Frenck-Mestre & Vaid, 1993;Lotus Lin et al., 2019;Marsh & Maki, 1976;Salillas & Wicha, 2012;Spelke & Tsivkin, 2001;Tamamaki, 1993;Van Rinsveld et al., 2016,^2017^), but not always (seeCerda et al., 2022;Tamamaki, 1993). Increasing evidence from the bilingualism literature suggests that bilinguals have language networks that are interconnected and highly interactive (Dijkstra & van Heuven, 2002;Hermans et al., 1998;Thierry & Wu, 2007). However, some argue that math information is encoded and/or processed in the brain in a language-specific manner (Campbell, 1994;Campbell & Clark, 1992;Campbell & Epp, 2004;Dehaene et al., 1999;Spelke & Tsivkin, 2001). No study has yet revealed the underlying neurocognitive mechanisms explaining simple multiplication processing in bilinguals. The goal of the current study was to fill this gap in the literature by determining if balanced, Spanish-English bilingual adults engage shared or separate cortical regions to process memorized multiplication facts across their languages.

Because multiplication facts are typically learned through rote memorization, it is thought that adults solve multiplication problems primarily through verbal memory retrieval, though they also engage in back-up strategies like calculation, transformation, or decomposition with problems that are more difficult to retrieve (Campbell & Fugelsang, 2001;Campbell & Timm, 2000;Campbell & Xue, 2001;Hecht, 1999,^2002^;LeFevre, Bisanz, et al., 1996;LeFevre, Sadesky, et al., 1996;Siegler, 1987). For example, smaller problems (e.g., 2 × 3) are more practiced and retrieved more efficiently than large problems (e.g., 8 × 9). This difference in speed and accuracy between small and large problems is known as the problem size effect and has been observed for all types of simple arithmetic operations, including multiplication (seeZbrodoff & Logan, 2004for a review).

In monolinguals, the left superior and middle temporal gyri (STG/MTG) have been associated with representing memorized arithmetic facts, along with the inferior frontal gyrus (IFG) in retrieval of these facts from long-term memory (Prado et al., 2011). Critically, STG/MTG and IFG are also implicated in aspects of language processing (Booth et al., 2002,^2003^,^2004^;Pollack & Ashby, 2018;Prado et al., 2011,^2014^). The STG/MTG become more active as children receive more years of math instruction, suggesting that they rely more on the retrieval of these facts from memory (Prado et al., 2014). Conversely, the IFG decreases activation with more years of math instruction, suggesting a more automatic retrieval of these facts with practice (Prado et al., 2014). Moreover, this area shows more activation when the task requires the retrieval of less practiced facts, such as large multiplication problems, even in adults (Prado et al., 2011). Additionally, evidence suggests that activation of the right intraparietal sulcus (IPS) increases with arithmetic task difficulty, where problems with an increasing number of operands engage IPS to a greater extent (Vaid & Menon, 2000). In line with this idea, problems that are more likely to involve strategies like decomposition, calculation, or transformation tend to engage IPS to a greater extent than problems that rely only on retrieval (Prado et al., 2013). The IPS is thought to be engaged during quantity processing in both adults and children (Arsalidou & Taylor, 2011;Ashkenazi et al., 2012;Chassy & Grodd, 2012;Kucian et al., 2008;Sokolowski et al., 2017). Although the engagement of these regions has been established in monolingual children and adults, it is unclear whether bilinguals engage these same regions in either or both of their languages.

The most prominent models of simple arithmetic processing imply that bilinguals use different or less efficient cognitive processes in the language not used for arithmetic learning (i.e., LA-). For example, the Triple Code Model (TCM) (Dehaene et al., 1999;Spelke & Tsivkin, 2001) suggests that simple arithmetic facts are stored as part of a learned lexicon of verbal associations in memory. The TCM proposes that arithmetic fact retrieval involves language areas not specific to numbers, including classic language areas of the left hemisphere (Dehaene & Cohen, 1995). The TCM does not explicitly address bilinguals, but in monolinguals, these verbal associations would by default only exist in the language in which the math concepts were originally learned. Taken together, this may imply that bilinguals engage separate cognitive processes for arithmetic across their languages, where direct retrieval occurs in LA+, and different processes are more likely engaged for LA- (e.g., translation; calculation-based strategies).

Determining the brain basis of behavioral differences in arithmetic performance across languages has proven challenging, especially because bilingual language background has not always been considered (seeCerda & Wicha, 2023for a review). Recent evidence suggests that language experience can mitigate language differences in the brain when processing arithmetic, even when bilinguals primarily learn math in one language (Cerda et al., 2019,^2022^,^2023^;Martinez-Lincoln et al., 2015). For example, in a recent study re-evaluating the LA+ advantage in bilingual arithmetic, when language proficiency and age of acquisition were equivalent across languages, there was no evidence that bilingual adults processed simple multiplication facts differently in LA+ and LA-, despite reporting a primary language of learning (Cerda et al., 2022).

Yet, there is evidence in the literature that even balanced bilinguals can show language differences, although subtle, when processing larger multiplication problems (Cerda et al., 2023).Cerda et al. (2023)recently used event-related potentials to investigate the problem size effect for examining the effects of language on bilingual arithmetic. The authors found a significant interaction between the language of arithmetic learning and problem size. This effect was driven by the fact that more difficult single-digit large problems in LA- were less efficiently processed than small problems in LA- and problems in LA+, regardless of size. The authors suggested that despite balanced language proficiency and early age of second language acquisition, increased frequency of use for one language for arithmetic (i.e., LA+) over a lifetime can drive subtle differences favoring that language, especially for large problems that are less frequently encountered in everyday life. Thus, it is possible that larger multiplication problems might differentially engage the brain areas typically observed during multiplication verification, particularly depending on the language the problems are presented in.

The current study aimed to investigate two questions in Spanish-English bilingual adults: 1) Does LA+ recruit STG/MTG to a greater extent than LA-, reflecting more robust verbal representations of multiplication facts in LA+? Because of weaker verbal representations in the temporal cortex for LA-, does solving multiplication problems in LA- require more effortful retrieval (left IFG) or the reliance on back-up quantity processes (bilateral IPS)? 2) Is there an interaction between language and problem size, where language differences are more pronounced for less practiced, large multiplication problems in comparison to more familiar, small problems? We hypothesize that language effects (i.e., LA+ > LA-) within STG/MTG would be most apparent for more frequently encountered small problems as compared to large problems. This would reflect greater reliance on verbal representations of arithmetic facts in LA+ in comparison to LA-. Additionally, we predict that language effects within IFG and IPS (i.e., LA- > LA+) would be most apparent for large problems as compared to small problems. This would reflect greater reliance on effortful retrieval of facts or greater reliance on back-up quantity processes for LA- in comparison to LA+.

## 2 Methods

The study hypotheses and analytical plan were preregistered through Open Science Framework prior to beginning data analyses (seehttps://osf.io/vbtr3). After completion of the original planned analyses, the preregistration was updated to include subsequent exploratory analyses.

### 2.1 Participants

Thirty-five Spanish-English bilingual adults were recruited for this study, with 5 from the University of Texas at Austin and 30 from the University of Houston. Informed written consent was obtained from all participants for being included in the study, approved by the Institutional Review Board of the University of Texas at San Antonio. To control for the effects of language proficiency and age of language acquisition, only bilinguals who learned both languages before 9 years old with equivalent proficiency across languages were included in the analyses. Language proficiency was based on comparisons of standardized language measures in English and Spanish, as described below.

Six of the initial 35 participants were excluded from the final sample (1 from the University of Texas at Austin and 5 from the University of Houston). Two of these participants were excluded due to failure to complete all experimental tasks, two participants due to excessive movement in the scanner (seefMRI data analysis sectionbelow), one participant due to unbalanced language proficiency, and one participant due to taking psychoactive medications at the time of testing. The final sample comprised 29 participants.

Participants were right-handed (assessed by the Edinburgh Inventory,Oldfield, 1971; range: 0.35-1.00), had normal or corrected-to-normal vision, normal hearing, no history of cognitive or perceptual deficits, were not diagnosed with language delays or learning disabilities, and were not taking medication that affected their cognition at the time of testing. All participants reported Spanish as their native language (L1), with 8 participants also reporting English as a simultaneous native language. Critically, L1 was not always equivalent to the language of learning multiplication (LA+). Eleven individuals learned multiplication in Spanish, and 18 reported learning multiplication in English. All participants reported using English as their current preferred math language, with 2 individuals additionally reporting to prefer Spanish depending on the context. All participants performed higher than chance (i.e., 50%) on small problems in the multiplication task in both languages. Additionally, they had no response bias in either the multiplication or the picture-word verification tasks (see below for more details), defined as no greater than 50% accuracy difference between true and false problems in the multiplication task, or matched and mismatched trials in the picture-word task.

### 2.2 Standardized measures

Language proficiency in English and Spanish was determined using two subtests (Test 14: Picture Vocabulary and Test 15: Oral Comprehension) of the Woodcock-Johnson Tests of Achievement (Woodcock, McGrew, et al., 2001) and one subtest (Test 8: Incomplete words) of the Woodcock-Johnson Tests of Cognitive Abilities (Woodcock, Mather, et al., 2001). Equivalent versions of these tests in Spanish were administered from the Batería III Woodcock-Munoz (Muñoz-Sandoval et al., 2005). These tests required participants to demonstrate vocabulary, language comprehension, and phonological awareness in each language. SeeTable 1for a summary of language measures. Aged-normalized classifications were obtained from the Woodcock-Johnson Proficiency Battery (WJPB-R) based on a participant’s raw assessment scores in each language. These classifications were then compared across each of the parallel subtests from the WJPB-R and the Batería III. Participants were considered to have balanced proficiency if their scores were within +/- 1 classification between languages on at least 2 out of the 3 subtests. This method has been used in previous investigations of arithmetic processing (seeCerda et al., 2019,^2022^,^2023^). It is important to note that although scores in English and Spanish were significantly different within the Picture Vocabulary and Incomplete Words subtests, all our participants still were classified as “balanced” based on our criteria above. Participants only needed to have comparable scores in 2 of the 3 subtests, and differences were driven by different people within each subtest.

**Table 1.** Language proficiency measures for the final sample (n = 29).

	Mean standardized score	SD	SEM	Sig
Picture Vocabulary				
English	94.1	(8.9)	1.7	* *p* < 0.01
Spanish	84.6	(12.2)	2.3	
Oral Comprehension				
English	100.5	(5.7)	1.1	*p* = 0.27
Spanish	98.1	(11.2)	2.1	
Incomplete Words				
English	98.3	(12.9)	2.4	* *p* < 0.01
Spanish	88.2	(8.1)	1.5	

Mean standardized scores, standard deviation (SD), and standard error of the mean (SEM) for the three subtests of the Woodcock Johnson (English) and Bateria III (Spanish). The column labeled “Sig” contains the*p*-value for a paired-samples t-test comparing scores across languages. The asterisk reflects a significant test,*p*< 0.05.

Multiplication fluency was measured using the Math Fluency: Multiplication subtest of the Wechsler Individual Achievement Test (WIAT) (Wechsler, 1992). This subtest asked participants to answer as many single-digit multiplication problems as possible in 60 seconds. Participants’ standardized scores ranged from 123 to 178, where 24 participants had average multiplication skills, 2 had superior multiplication skills, and 3 had low multiplication skills according to average young adult multiplication performance. A measure of working memory was collected for use outside the scope of the current research questions.

### 2.3 Scanner tasks

Participants performed two localizer tasks (verbal and numerosity) and the experimental task (single-digit multiplication verification) in a single experimental session. The verbal localizer task and the experimental task were administered in both English and Spanish, alternating language order across participants. The verbal localizer task was used as a functional localizer for temporal-frontal areas in each language, including the left superior and middle temporal gyri (STG/MTG) and the left inferior frontal gyrus (IFG). The numerosity localizer was used as a functional localizer of quantity processing regions in the bilateral intraparietal sulci (IPS). All tasks also included control trials in which a blue square was presented, and participants were asked to press a response button with their index finger when the box turned red.

#### 2.3.1 Single-digit multiplication verification task

Participants were presented with two runs of single-digit multiplication problems in English and two runs in Spanish (see sample trial inFig. 1). This task was adapted from previous tasks using Arabic Digits to study arithmetic processing (seeBerteletti et al., 2014;Demir-Lira et al., 2020;Prado et al., 2011,^2013^,^2014^;Suárez‐Pellicioni & Booth, 2022;Suárez-Pellicioni et al., 2020,^2021^) to allow for auditory presentation of the problems. Only two other fMRI studies on bilingual arithmetic have used auditory presentation of arithmetic problems (Van Rinsveld et al., 2017;Wang et al., 2007).Van Rinsveld et al. (2017)presented bilinguals with simple and complex addition problems, andWang et al. (2007)presented second language learners with complex multiplication problems involving two-digit numbers. However, the current study is the first to use auditory presentation of simple multiplication problems across a bilingual’s languages while acquiring images with fMRI.

**Fig. 1.**
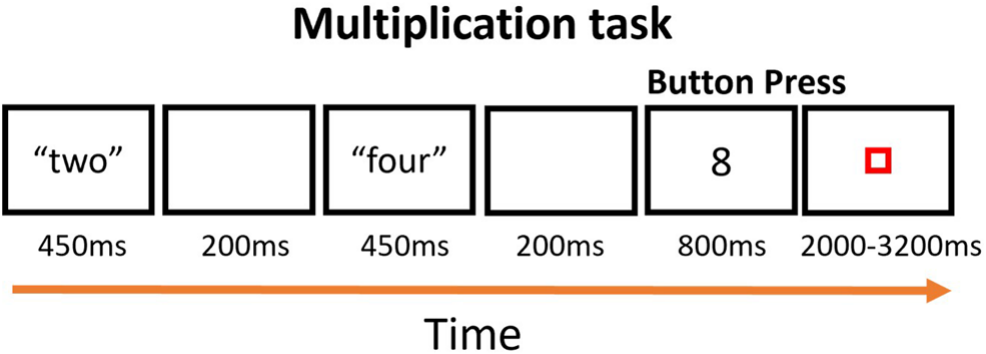
Sample trial of the multiplication task in English. The Spanish task was identical except for the number operands, which were spoken in Spanish (“dos” “cuatro”). Participants did not see any written words. All words were normalized to 450 ms in duration.

For this task, participants heard two spoken number words, followed by a visual Arabic digit, and the task was to determine whether the Arabic digit was the correct product of the two spoken numbers (i.e., determine if the problem was true or false). Behavioral responses were recorded using an MR-compatible keypad, and participants responded with their right hand. Participants used their index finger if the proposed solution was true and their middle finger if the proposed solution was false. We deliberately used spoken number words for problem operands to ensure that participants engaged in either language when performing the multiplication task. Each language was presented in separate runs with language-specific instructions to avoid language switching (seeGrosjean, 1998). Language order was reversed in half of the participants. Proposed solutions were presented as an Arabic digit for all trials in both languages, so the effects could be attributed to the language used to access the math facts from memory. Visual stimuli were projected onto a screen that was viewed by the participants through a mirror attached to the MRI head-coil, and the audio was presented using Siemens MRI-compatible insert headphones.

Thirty-six single-digit multiplication problems were presented per run and were classified based on problem size. Problems with correct solutions smaller than 25 were considered small problems, and problems with correct solutions larger than 25 were considered large problems. This classification of problem size was chosen for consistency with previous arithmetic studies in both bilingual and monolingual populations (Cerda et al., 2023;Dickson and Wicha, 2019;Dickson, Grenier, Obinyan, and Wicha, 2022). Multiplication trials were divided as follows: 12 small problems with true solutions, 12 large problems with true solutions, 6 small problems with false solutions, and 6 large problems with false solutions. To ensure equivalent task difficulty across languages, identical sets of core multiplication problems were presented as spoken number words in English and Spanish, in a different semi-randomized order of trials.

Additionally, this task included 12 control trials per run. For these trials, participants saw a blue square and were instructed to press a button with their index finger when the blue square turned red (seeFig. 2C). With the inclusion of these trials, the duration of each run was approximately 6 minutes.

**Fig. 2.**
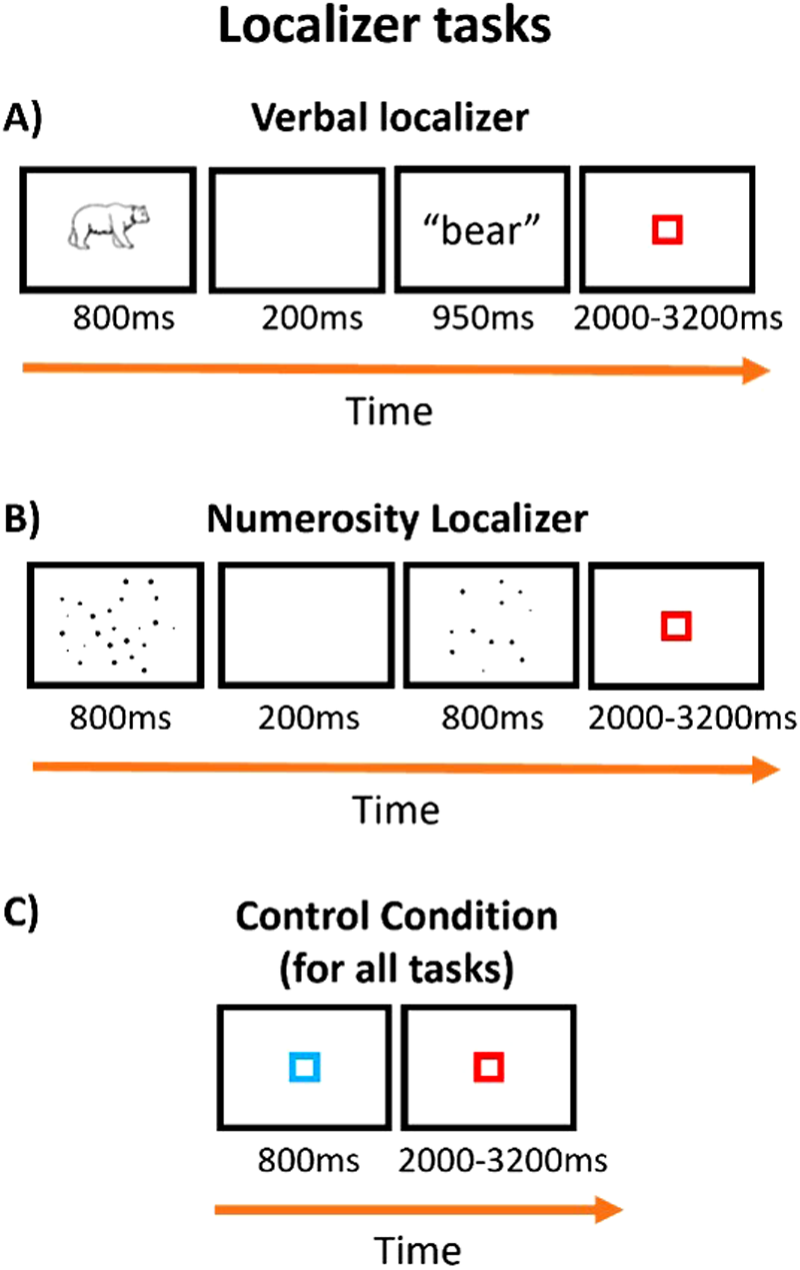
(A) Sample trial of the English Verbal Localizer. Trial structure was identical for the Spanish Verbal Localizer, with Spanish spoken words. (B) Sample trial of the Numerosity Localizer. (C) Example of the control trial used throughout all tasks.

#### 2.3.2 Verbal localizer: picture-word verification task

Participants completed two runs of the picture-word verification task in English and two runs in Spanish. Language order assignments for each participant were the same as the language order of the multiplication task (e.g., starting both tasks in Spanish). During the task, participants saw a black-and-white line drawing of an object followed by a spoken word and determined whether the word matched the presented picture or not (see sample trial in English inFig. 2A). Identical to the multiplication verification task, behavioral responses were also recorded using an MR-compatible keypad and participants responded with their right hand. Participants used their index finger if the picture-word pairs matched and their middle finger if they did not match. Mismatched trials were different across languages to avoid predictability (Task in English:*pig/butterfly*; Task in Spanish:*cerdo/tigre*(meaning pig/tiger), and words were matched on frequency and animacy (animate/inanimate object) across languages and conditions (seeCerda et al., 2019for details on stimulus construction). Forty experimental trials were presented per run, with an equal number of matched and mismatched trials. Additionally, 13 control trials were included in this task per run (Fig. 2C), which were identical to the control trials included in the multiplication task. The duration of each run was approximately 6 minutes.

#### 2.3.3 Numerosity localizer: dot comparison

Participants completed two runs of the dot comparison task and were tasked with determining which of two sequentially presented dot arrays contained more dots (Fig. 2B). This task has been used previously as a localizer of quantity regions in the parietal cortex (Berteletti et al., 2014;Prado et al., 2014;Suárez-Pellicioni & Booth, 2018;Suárez-Pellicioni et al., 2020). Thirty-six experimental trials were divided as follows: 12 easy comparisons (i.e., a ratio of 1:3; e.g., 12 dots to 36 dots), 12 medium comparisons (i.e., a ratio of 1:2; e.g., 18 dots to 36 dots), and 12 hard comparisons (i.e., a ratio of 2:3; e.g., 24 dots to 36 dots). Dot size was controlled across arrays to ensure that participants were judging differences in the number of dots, rather than the surface area covered by the dots. Additionally, 12 control trials (Fig. 2C), identical to the control trials included in the multiplication task, were presented per run. The duration of each run was approximately 3 minutes.

#### 2.3.4 Stimulus timing

For the multiplication task, all spoken number words in both English and Spanish were normalized to 450 ms duration, which was chosen specifically to reduce distortion in both languages. Experimental trials began with the presentation of the first operand (450 ms) followed by a 200 ms interstimulus interval (ISI) before the second operand (450 ms). During this time, participants saw a blank white screen. After another 200 ms ISI, an Arabic Digit solution appeared on the screen for 800 ms. All problems were followed by a red fixation square, presented with a variable duration (2,000 ms, 2,600 ms, or 3,200 ms; 1,200 ms jitter) to help with convolution of each trial. Each run ended with 22 seconds of passive visual fixation to also aid in deconvolution of the final trials.

Stimulus timing was similar for both localizer tasks. Trials began with the presentation of the first stimulus (i.e., line drawing or first dot array, depending on the task) for 800 ms, followed by a blank screen for 200 ms. For the verbal localizer, the second stimulus was a spoken word in English or Spanish, across separate blocks, presented for up to 950 ms. For the numerosity localizer, the second stimulus was the second dot array, presented for 800 ms. For both tasks the second stimulus was followed by a red fixation square, presented with variable timing (2,000 ms, 2,600 ms, or 3,200 ms; 1,200 ms jitter) to help with convolution. Participants were instructed to answer as quickly and accurately as possible as soon as they knew the answer. The timing of the task was identical across all participants. Each run ended with 22 seconds of passive visual fixation for deconvolution of the final trials.

### 2.4 fMRI data acquisition

Images were collected using a Siemens Prisma Fit scanner (3T) at the Baylor College of Medicine’s Core for Advanced Magnetic Resonance Imaging (Houston, Texas) and a Siemens Skyra scanner (3T) at the University of Texas’s Biomedical Imaging Center (Austin, Texas). The fMRI blood oxygenation level dependent (BOLD) signal was measured with a susceptibility-weighted single-shot echo planar imaging (EPI) sequence. The following parameters were used across both scanners to ensure comparability of the datasets: TE = 30 ms, flip angle = 68 s, matrix size = 256 x 256, field of view = 256, slice thickness = 2 mm, number of slices = 64, TR = 1,250 ms, and voxel size: 2 x 2 x 2 mm. Before functional image acquisition, a high-resolution T1-weighted 3D structural image was acquired for each participant with the following parameters: TR = 1,900 ms, TE = 2.43 ms, matrix size = 256 x 256, field of view = 256 mm, slice thickness = 1 mm, and number of slices = 192.

### 2.5 fMRI data analysis

#### 2.5.1 Preprocessing

Preprocessing and analysis of the fMRI data was computed using Statistical Parametric Mapping (SPM12http://www.fil.ion.ucl.ac.uk/spm). First, all functional images were realigned to their mean functional image across runs. An anatomical brain mask was created by combining the segmentation products (i.e., grey matter, white matter, and cerebrospinal fluid), and then applied to its original anatomical image to produce a skull-stripped anatomical image. Then, the mean functional image and all functional images were co-registered to the skull-stripped anatomical image. After normalization, all the functional images were normalized to the standard T1 Montreal Neurological Institute (MNI) template. Afterward, smoothing was applied to all the functional images with a 6 mm isotropic Gaussian kernel. All coordinates are reported in MNI space for consistency with previous fMRI studies in math cognition (e.g.,Berteletti et al., 2014;Prado et al., 2011,^2013^,^2014^;Suárez‐Pellicioni & Booth, 2022;Suárez-Pellicioni et al., 2020).

To reduce movement effects on the brain signal, Art-Repair (http://cibsr.stanford.edu/tools/human-brain-project/artrepair-software.html) was used to identify outlier volumes. Outliers were defined as volume-to-volume head movement exceeding 1.5 mm in any direction, head movement greater than 5 mm in any direction from the mean functional image across runs, or deviations of more than 4% from the mean global signal intensity. Outlier volumes were repaired by interpolation between the nearest non-outlier volumes. Participants included in the analysis had no more than 10% of the total volumes repaired and no more than 6 consecutive volumes repaired in each run. Six motion parameters estimated during realignment were entered in the first-level modeling as regressors, and the repaired volumes were deweighted (Mazaika et al., 2009).

#### 2.5.2 fMRI processing

Event-related statistical analysis was performed according to the general linear model (GLM). Activation was modeled as epochs with onsets time-locked to the presentation of the first stimulus in each trial (i.e., first spoken operand of the multiplication problem, line drawing of the verbal localizer, and first dot array in the numerosity localizer). To equate for power in the analysis, all proposed solutions (i.e., true and false) and all participants’ responses (correct and incorrect) were included in the model. All epochs were convolved with a canonical hemodynamic response function. The time series data were high-pass filtered (1/128 Hz), and serial correlations were corrected using an autoregressive AR model.

#### 2.5.3 Defining regions of interest (ROIs)

Regions of interest were constructed by constraining brain activation from the two localizer tasks within anatomically defined regions in the left superior and middle temporal gyri (STG/MTG), the left inferior frontal gyrus (IFG), and the bilateral intraparietal sulci (IPS). These anatomical regions were defined using the automated anatomical labeling (AAL) template (part of the Wfupickatlas tool). Template regions of left STG and left MTG were combined into a single anatomical mask. The IFG anatomical mask included the left pars opercularis, left pars trangularis, and left pars orbitalis. The anatomical mask for IPS was constructed by dilating (3D dilation of 2) both the inferior and superior parietal lobules and isolating the intersection between the two regions, as previously done bySuárez-Pellicioni and Booth (2018). This method was used to create separate ROIs in the left and right IPS.

In order to define combined (i.e., functional and anatomically defined) ROIs, these anatomical regions were used to constrain the brain activity from the localizer tasks. More specifically, verbal ROIs were defined by constraining brain activation during the verbal localizer tasks separately for each language (i.e., “picture-word pairs in Spanish vs. control” and “picture-word pairs in English vs. control” contrasts) within the left STG/MTG and left IFG. We then extracted the top 25% of voxels showing maximal activation (regardless of significance) for each contrast. The union of the top voxels across the two languages constituted our combined verbal ROI. Quantity ROIs were defined by constraining brain activation during the numerosity localizer task (i.e., “dot array pairs vs. control” contrast) within the left and right IPS. We then extracted the top 25% voxels showing maximal activation (regardless of significance) within the left and right hemisphere, which constituted our two quantity ROIs.

#### 2.5.4 Multiplication task analysis

For the multiplication task, a first-level contrast of “all problems greater than control” was performed for each participant and each language. Then, within each of the three ROIs (STG/MTG, IFG, and IPS), we compared brain activation elicited by the multiplication task across the two languages (language of learning vs. the other language, or LA+ vs. LA-). For question 1, we compared the languages collapsed across problem size (i.e., “LA+ [all problems – control] > LA- [all problems – control]” and “LA- [all problems – control] > LA+ [all problems – control].”) For question 2, we tested a language (LA+, LA-) by problem size (small, large) interaction within our defined ROIs. This was done using the following contrasts: LA+ [small problems – large problems] > LA- [small problems – large problems] and LA- [large problems – small problems] > LA+ [large problems – small problems].

Significance thresholds for the Multiplication Task within the ROIs were determined using 3dClustSim (December 2015; seehttp://afni.nimh.nih.gov/). This approach calculates the threshold for significant cluster sizes that would be unlikely to occur by chance within a masked brain volume at a specified uncorrected alpha level (for an example of how this approach has been applied to a similar dataset, seeSuárez‐Pellicioni & Booth, 2022). Using 3dClustSim, we carried out ten thousand Monte Carlo simulations of random noise activations using a cluster-wise probability threshold of 0.05 and a voxel-wise threshold of 0.005 within each of our ROIs. The number of simulations in which clusters of different sizes appear within each ROI mask was tallied, and these data were used to calculate cluster size thresholds for significance. Additionally, we used 3dFWHMx to calculate the smoothness of the data for every single participant, using a spatial autocorrelation function. These smoothness values were averaged across all participants and entered into 3dClustSim to calculate the cluster size needed for significance for a given ROI. Clusters exceeding these size thresholds were considered significant. Based on this calculation, a cluster size of 45, 40, 15, and 13 voxels was needed for significance for the left STG/MTG, left IFG, left IPS, and right IPS ROIs, respectively (ACF values = 0.32, 5.80, 21.00). Additionally, a cluster size of 732 voxels was needed for significance at the whole-brain level.

## 3 Results

### 3.1 Multiplication tasks: behavioral results

First, we examined participants’ performance (accuracy and response time) on the multiplication task in their language of learning (LA+) and in the other language (LA-). Participant averages of accuracy and response time were extracted separately from the conditions of interest based on a within-subject repeated-measures design, including Problem size (Small, Large), Language (i.e., language of learning: LA+, LA-), and Correctness (i.e., the correctness of the proposed solution: true, false). To determine the contribution of individual differences on behavioral effects, linear mixed models were created using a generalized linear model from the “lme4” package in R (Bates et al., 2015), with Subjects as a random effect. Satterthwaite’s method was used to estimate denominator degrees of freedom for F statistics. Only trials with accurate responses were included in response time analyses. For transparency, note that performance data were originally analyzed using a repeated-measures analysis of variance, including Language, Problem size, and Correctness as within-subject factors. This original analysis of accuracy data revealed a different pattern of results. However, per reviewer’s suggestion, we analyzed the data using linear mixed models.

#### 3.1.1 Task accuracy

For our analysis of Accuracy (seeFig. 3), there was a main effect of Problem Size (*F*(1, 196) = 140.55;*p*< 0.001; η_p_^2^= 0.42) with greater accuracy for small (97.45%; SE = 0.63) than large problems (80.53%; SE = 2.83). A main effect of Correctness (*F*(1, 196) = 31.50;*p*< 0.001; η_p_^2^= 0.14) revealed higher accuracy for true (92.99%; SE = 1.203) than false (84.986; SE = 2.252) problems. There was a trend for a main effect of Language (*F*(1, 196) = 3.01;*p*= 0.083) suggesting that participants tended to be more accurate at responding to problems in LA+ (90.23%; SE = 1.521) in comparison to LA- (87.75%; SE = 1.815). However, this pattern should be interpreted with caution, as it did not reach significance. An interaction between Problem Size and Correctness was significant (*F*(1, 196) = 18.96;*p*< 0.001; η_p_^2^= 0.09). This interaction was driven by responses to large problems, with higher accuracy in verifying true solutions (M = 87.6; SE = 2.03) compared to false solutions (M = 73.4; SE = 2.03;*t*(196) = 7.04;*p*< 0.001). There was no significant difference in verifying true and false solutions for small problems (*t*(196) = 0.89;*p*> 0.1). No other interactions were significant (i.e.,*p*> 0.05).

**Fig. 3.**
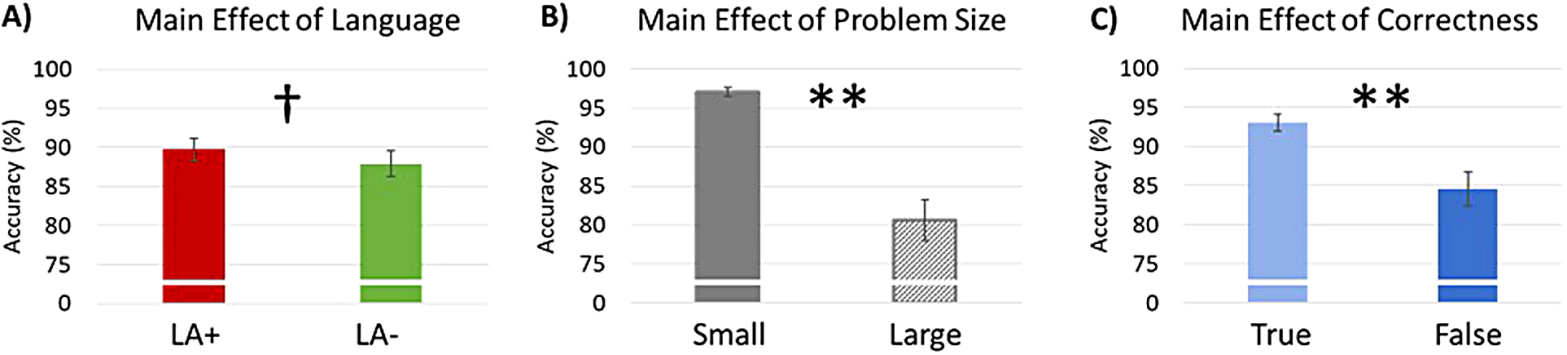
Multiplication Task Accuracy. Bar charts depicting task accuracy for the multiplication verification task. Charts on the top panel depict the main effects of (A) Language, (B) Problem size, and (C) Correctness. The language of learning multiplication (LA+) is depicted in red, and the other language (LA-) is depicted in green. Error bars represent standard error of the mean. †*p*< 0.10; **p< 0.001.

#### 3.1.2 Task response time

For Response Time (seeFig. 4), there was a main effect of Language (*F*(1, 196) = 4.54;*p*= 0.034; η_p_^2^= 0.02) with slower responses to problems in LA+ (1,052.94 ms; SE = 50.69) than LA- (1,012.84 ms; SE = 53.83). As expected, there was a main effect of Problem size (*F*(1, 196) = 226.46;*p*< 0.001; η_p_^2^= 0.54) where responses were faster for small (891.43 ms; SE = 54.59) than large problems (1,174.34; SE = 51.85). There was also a main effect of Correctness (*F*(1, 196) = 93.46;*p*< 0.001; η_p_^2^= 0.24), driven by faster responses to true (942.01 ms; SE = 53.30) than false (1,123.76 ms; SE = 51.44) problems. The interaction between Language and Problem size was significant (*F*(1, 196) = 6.89;*p*< 0.01; η_p_^2^= 0.03). This effect was driven by a larger problem size effect for LA+ than LA-, with participants responding more slowly to large problems in LA+ than LA- (*t*(196) = 3.36;*p*< 0.001). There was no evidence of language-related difference for small problems (*t*(196) = -0.34;*p*> 0.1). No other interactions were significant (i.e.,*p*> 0.05).

**Fig. 4.**
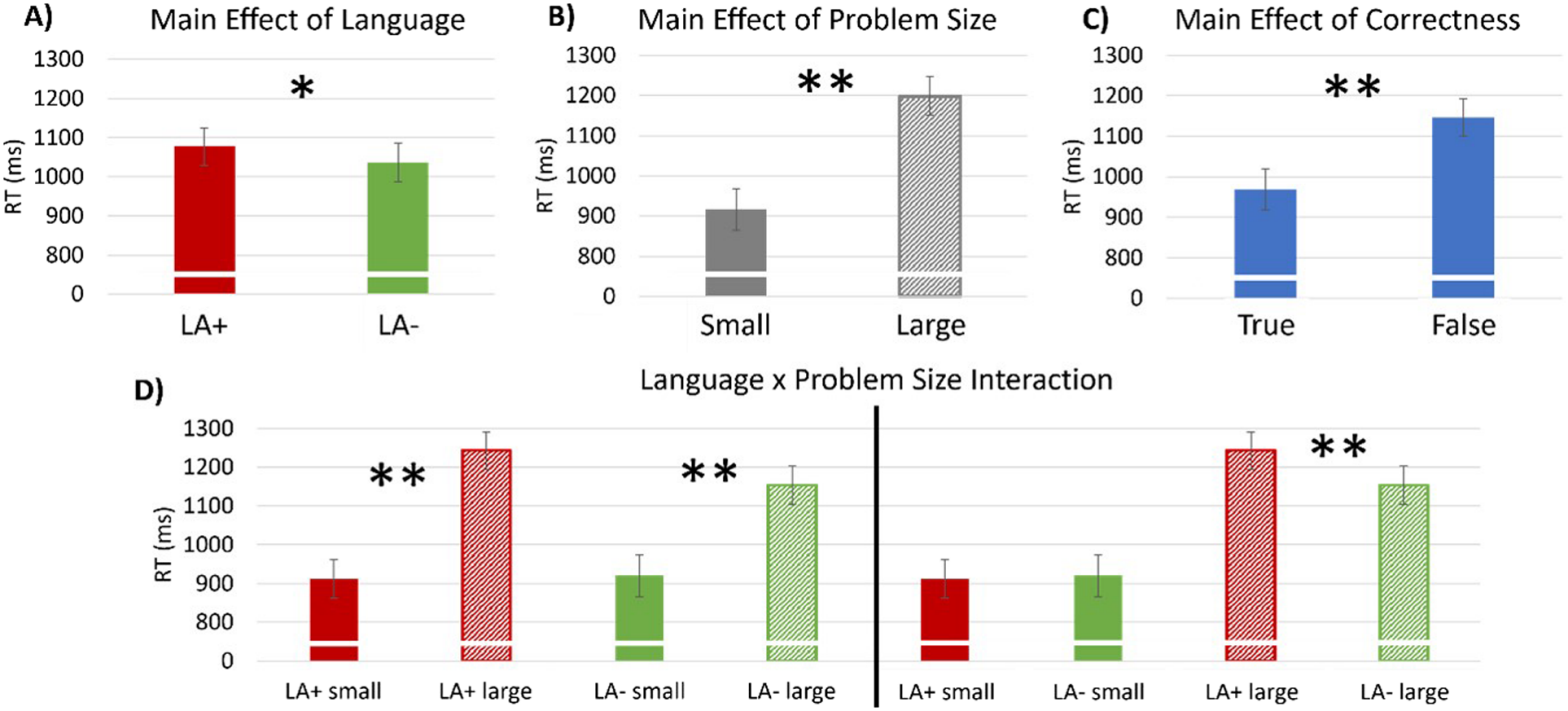
Multiplication Task Response Time. Bar charts depicting response time for the multiplication verification task by condition. Charts on the top panel depict the main effects of (A) Language, (B) Problem size, and (C) Correctness. The language of learning multiplication (LA+) is depicted in red, and the other language (LA-) is depicted in green. The bottom panel (D) shows significant problem size (small vs. large) differences for both LA+ and LA- and significant language differences (LA+ vs. LA-) for large problems. Bars on both sides of the black dividing bar represent identical data, reordered to highlight differences across conditions. Error bars represent standard error of the mean. *p< 0.05; **p< 0.001.

#### 3.1.3 Summary of behavioral results

In summary, our sample of balanced bilingual adults verified the correctness of small multiplication problems faster and more accurately than large problems. This finding was expected based on the typical problem size effect reported in the literature (seeZbrodoff & Logan, 2004for review). Additionally, these adults showed differences in the speed of responding to problems across languages, but no significant differences in their accuracy across languages. Participants were slower to verify the correctness of problems presented in LA+ compared to LA-. An interaction between Problem size and Language was also observed for response time, driven by the larger, more difficult problems. Participants responded to large problems in LA- more quickly than problems in LA+.

### 3.2 fMRI results

#### 3.2.1 Defining regions of interest (ROIs) using localizer tasks

**Fig. 5.**
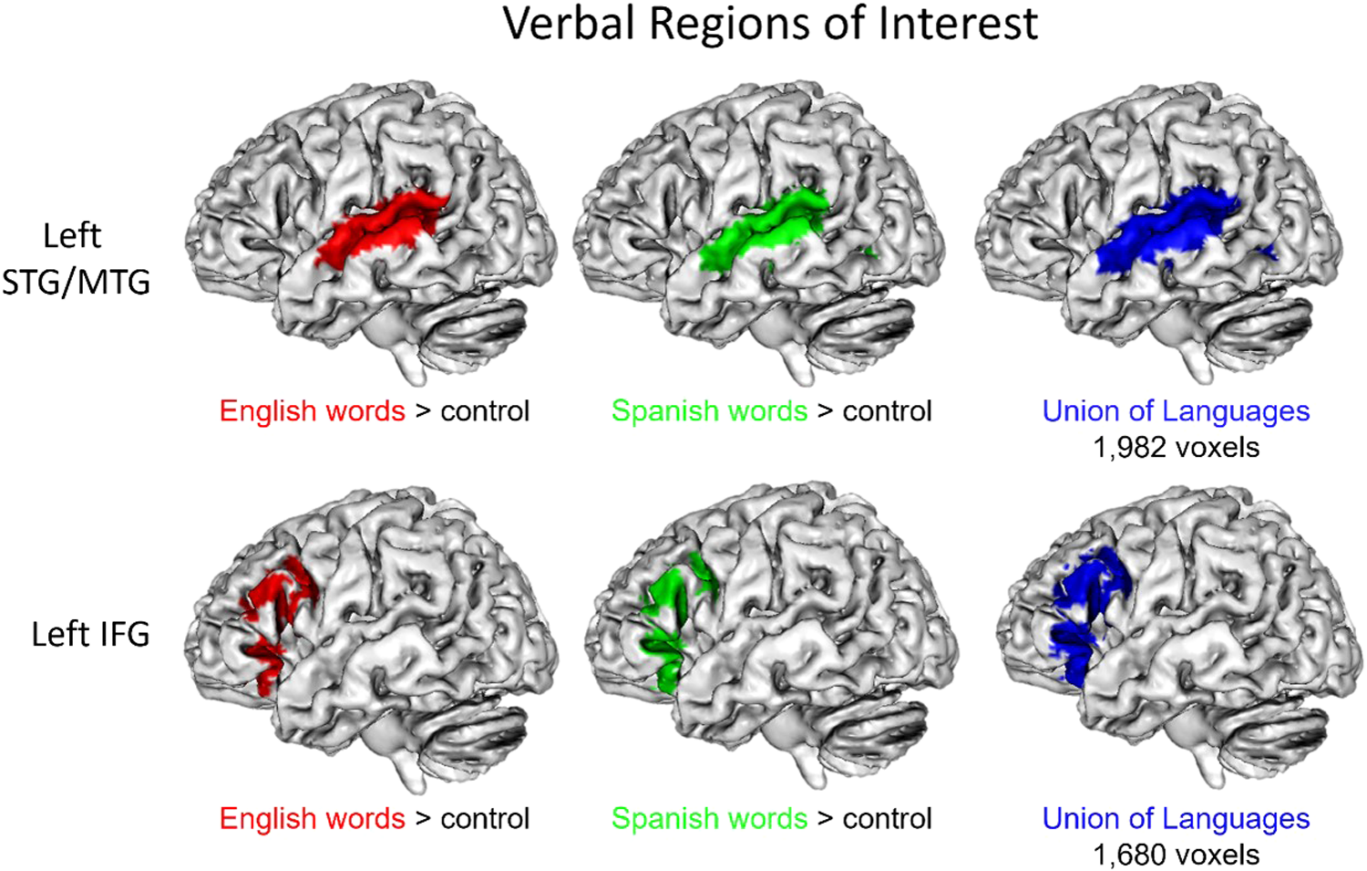
Regions of interest identified using the verbal localizer task. 25% of voxels showing maximal activation (regardless of significance) within left STG/MTG (top panel) and within left IFG (bottom panel) for the contrast of English words greater than control (shown in red in the left column) and the contrast of Spanish words greater than control (shown in green in the middle column). The right column shows, in blue, the union of the extracted voxels across the two languages in temporal (top panel) and frontal (bottom panel) cortices.

As described in the methods, the verbal localizer task was used to identify language regions in temporal and frontal cortices, and the numerosity localizer was used to identify quantity processing regions in the parietal cortex. Within these regions of interest, we assessed activation patterns for the multiplication task, making direct comparisons across languages.Figure 5depicts the 25% of voxels showing maximal activation (regardless of significance) within our regions of interest for the verbal localizer task. As described above, the union of the top voxels across the two languages constituted our combined verbal ROI. The resulting left MTG/STG and left IFG ROIs comprised 1,982 voxels and 1,680 voxels, respectively.

Quantity ROIs were defined using the contrast of all dots > control within the anatomical right and left IPS, separately. We extracted the 25% of voxels showing maximal activation (regardless of significance), which constituted our combined quantity ROI (seeFig. 6). The resulting ROIs for right and left IPS were 428 voxels and 547 voxels, respectively.

**Fig. 6.**
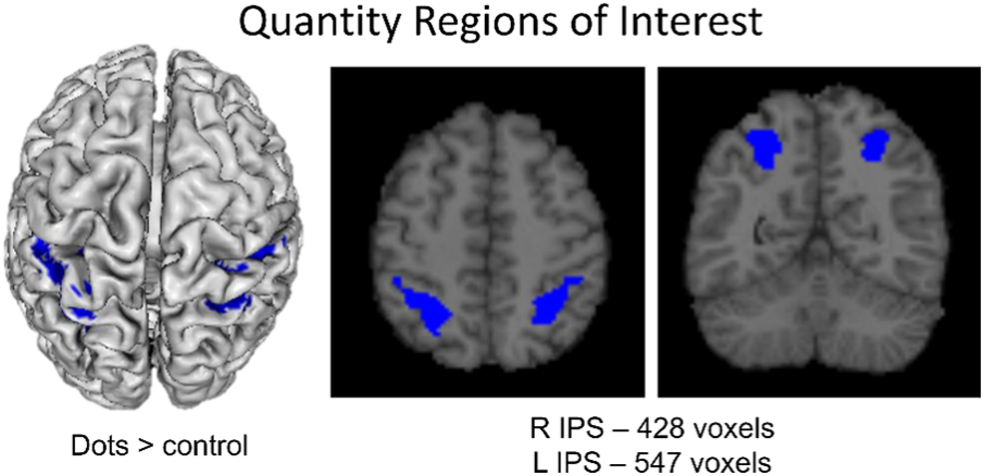
Regions of interest identified using the quantity localizer task. The voxels in blue depict the 25% of voxels showing maximal activation (regardless of significance) for the contrast “all dots vs. control” within the bilateral IPS.

#### 3.2.2 Multiplication tasks

##### 3.2.2.1 ROI analyses

The analysis carried out to answer question 1 included multiplication problems collapsed across problem size and correctness to make direct language comparisons within our regions of interest. This analysis revealed no significant voxels for the contrast LA+ > LA- or the reverse contrast, LA- > LA+. For question 2, the analysis testing a language (LA+, LA-) by problem size (small, large) interaction also revealed no significant clusters.

##### 3.2.2.2 Exploratory whole-brain analysis

To determine if there were effects outside of our ROIs, we conducted an exploratory analysis of the whole brain for both questions 1 and 2. We did not find any significant clusters for the contrast LA+ > LA- or the reverse contrast. Additionally, we did not find any significant clusters reflecting an interaction between language and problem size.

##### 3.2.2.3 Exploratory analysis of the main effect of problem size

**Fig. 7.**
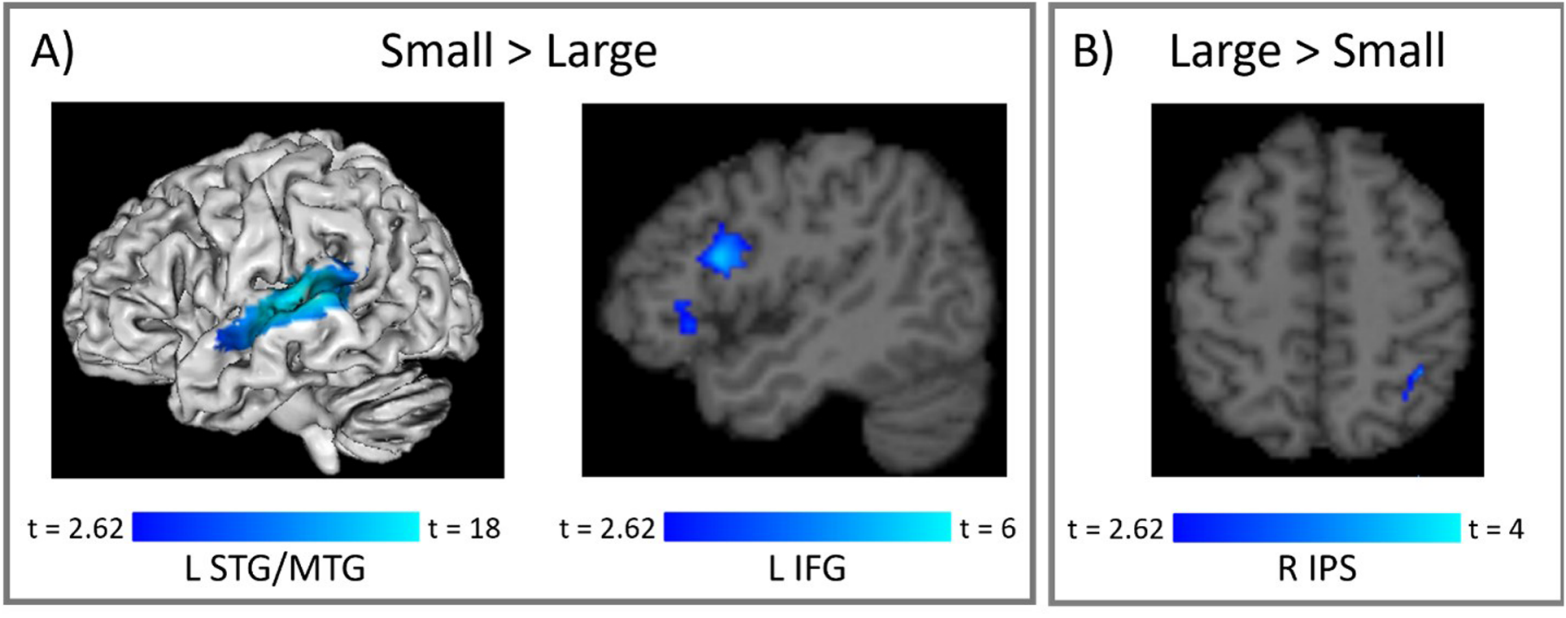
Main effects of problem size within the regions of interest. (A) Depiction of the brain activation for the contrast small > large for the multiplication task, collapsed across languages. The significant cluster in left STG/MTG is shown on a rendered brain on the left hemisphere. The significant clusters in the left IFG are shown on the right. (B) Depiction of the brain activation for the contrast large > small for the multiplication task, collapsed across languages, showing the significant clusters in the right IPS.

Although there was no significant interaction between language and problem size, an exploratory analysis revealed a main effect of problem size within the ROIs. For the contrast of small > large, there were significant clusters in the left STG/MTG and left IFG (seeFig. 7A). For the large > small contrast, there were 2 significant clusters in the right IPS (Fig. 7B). More specific information about these clusters is shown inTable 2. Moreover, an analysis at the whole-brain level confirmed the main effect of problem size, extending outside of our regions of interest. SeeTable 3for more specific information about these clusters.

**Table 2.** Cluster information for the main effect of problem size for the multiplication task within the regions of interest.

Anatomical location		MNI coordinates				
	*~BA*	*X*	*Y*	*Z*	*k*	*z* -value
Multiplication Task [small > large]						
Left STG/MTG	22/21	-60	-32	6	1,968	>8
Left IFG	44/45	-46-38	1426	204	387119	4.903.51
Multiplication Task [large > small]						
Right IPS	7	40	-50	54	46	3.30

Cluster size (k), MNI coordinates of the peaks, Z values, and approximate Brodmann areas (~BA) for the clusters showing significant activation for the problem size contrast.

**Table 3.** Cluster information for the main effect of problem size for the multiplication task at the whole-brain level.

Anatomical location		MNI Coordinates				
	*~BA*	*X*	*Y*	*Z*	*k*	*z* -value
Multiplication Task [small > large]						
Right Sup Temporal Gyrus	22	64	-20	4	5,627	>8
Left Sup Temporal Gyrus	22	-60	-32	-3	5,559	>8
Left Precentral Gyrus	4	-52	-6	48	1,333	7.61
Left Med Frontal Gyrus	6	-6	4	58	756	7.09
Right Mid Occipital Gyrus	18	20	-92	16	8,943	6.30
Multiplication Task [large > small]						
Right Inf Parietal Lob	40	56	-34	54	772	4.22

Cluster size (k), MNI coordinates of the peaks, Z values, and approximate Brodmann areas (~BA) for the clusters showing significant activation for the problem size contrasts.

#### 3.2.3 Summary of fMRI results

In summary, we did not observe significant differences in activation between LA+ and LA- in any of the regions of interest that we hypothesized (i.e., left STG/MTG, left IFG, and bilateral IPS) or at the whole-brain level. Similarly, we did not find any evidence of an interaction between Language and Problem size within these regions or at the whole-brain level. We did observe a main effect of Problem size (i.e., collapsed across languages), where small problems engaged left STG/MTG and left IFG to a greater extent than large problems and large problems engaged right IPS to a greater extent than small problems. The problem size effects within the left STG/MTG and right IPS are consistent with previous work (Prado et al., 2013;^2014^;Menon et al., 2000). However, we did not expect small problems to engage the left IFG to a greater extent than large problems. Typically, this region is associated with processes related to effortful retrieval of arithmetic facts (Prado et al., 2011). We discuss the broader implications of these results in the Discussion.

### 3.3 Additional exploratory analyses

#### 3.3.1 Effects of LA- vocabulary fluency

Evidence from the bilingual arithmetic literature suggests that increased language fluency in a weaker language can mitigate language differences for arithmetic processing (Garcia et al., 2021;Martinez-Lincoln et al., 2015;Tamamaki, 1993), so we performed additional exploratory analyses to determine if language proficiency in LA- was associated with behavioral performance and brain activation for multiplication processing.

We measured multiple dimensions of language fluency from our bilingual sample (i.e., picture vocabulary, oral comprehension, phonological awareness, self-reported fluency, etc.); however, based on prior work, we chose to use only one standardized measure of language fluency in these analyses. Previous bilingual language studies used the Boston Naming Test as a proxy for bilingual language fluency to investigate processing differences across languages in the brain (Hernandez et al., 2000;Moreno & Kutas, 2005;Salillas & Wicha, 2012). The Boston Naming Test requires participants to orally name a series of line drawings in each language. In the current study, we collected a similar measure using the Picture Vocabulary subtest of the Woodcock-Johnson Tests of Achievement (English) and the parallel Vocabulario Sobre Dibujos subtest of the Batería III (Spanish; seeStandardized Measures sectionabove).

First, we studied the effects of LA- language fluency (i.e., vocabulary fluency) on language-related differences (LA+ – LA-) in response time and accuracy in the arithmetic tasks. We predicted a negative relation between LA- vocabulary fluency and language-related differences in arithmetic performance, where lower LA- fluency will result in larger performance differences across languages. More specifically, we expected LA- fluency to be associated with poorer LA- performance on the multiplication task, particularly for large problems.

We conducted separate hierarchical regression analyses for accuracy and response times. LA+ vocabulary fluency was entered into the models as a covariate of no interest and LA- vocabulary fluency as the independent variable. The rationale for including LA+ as a covariate of no interest was to account for between-subject variability in general language skill. The dependent measure was the difference in task performance in multiplication across languages (i.e., LA+ accuracy – LA- accuracy or LA+ response time – LA- response time). These analyses did not reveal any significant relations between language differences in performance and LA- vocabulary fluency above and beyond LA+ vocabulary fluency (seeTable 4).

**Table 4.** Hierarchical models exploring the relation between LA- vocabulary fluency and language differences in arithmetic task performance.

		Small problems		Large problems	
*Model*	Predictors	R ^2^ Δ	*p* -value	R ^2^ Δ	*p* -value
Accuracy * (LA+ - LA-)*
*1*	LA+ Vocabulary Fluency				
*2*	LA+ Vocabulary Fluency LA- Vocabulary Fluency	0.030	>0.1	0.017	>0.1
***Response Time** (LA+ - LA-) *			
*1*	LA+ Vocabulary Fluency				
*2*	LA+ Vocabulary Fluency LA- Vocabulary Fluency	<0.001	>0.1	0.018	>0.1

For both Accuracy and Response Time (RT), language differences for performance on small problems and large problems were modeled separately. The additional variance explained, R^2^Δ, and corresponding*p*-values are shown.

Second, we completed a similar analysis using brain activation for the contrast LA- > LA+ for small and large problems separately within our regions of interest (left STG/MTG, left IFG, and bilateral IPS). Overall, we expected that lower LA- fluency would be associated with larger language-related differences within each ROI; however, we had separate predictions for what might be driving potential effects for each region. We predicted that lower levels of LA- vocabulary proficiency would be associated with less activation in STG/MTG for LA- multiplication (i.e., LA- > control). We predicted that this relation would be significant for both small and large problems (vs. control), though stronger for small problems (Prado et al., 2011,^2014^). In contrast, lower levels of LA- vocabulary proficiency might be associated with increased brain activation in the left IFG and right IPS for LA- multiplication (i.e., LA- > control). We predicted that this relation would be significant for both small and large problems, although stronger for larger problems (Prado et al., 2011,^2014^).

To address these hypotheses, a hierarchical regression analysis was performed with LA+ vocabulary fluency entered into the model as a covariate of no interest and LA- vocabulary fluency as the independent variable. The dependent measure was brain activation for the contrast LA- > LA+ for small problems and large problems separately within our ROIs. These analyses did not reveal any significant relation between language differences in brain activation and LA- vocabulary fluency above and beyond LA+ vocabulary fluency (seeTable 5).

**Table 5.** Hierarchical models exploring the relation between LA- vocabulary fluency and language differences in brain measures taken from our regions of interest, left STG/MTG, left IFG, and bilateral IPS.

		Small problems		Large problems	
*Model*	Predictors	R ^2^ Δ	*p* -value	R ^2^ Δ	*p* -value
***Left STG/MTG***
*1*	LA+ Vocabulary Fluency				
*2*	LA+ Vocabulary FluencyLA- Vocabulary Fluency	0.013	>0.1	0.013	>0.1
***Left IFG***					
*1*	LA+ Vocabulary Fluency				
*2*	LA+ Vocabulary FluencyLA- Vocabulary Fluency	0.040	>0.1	<0.001	>0.1
***Left IPS***					
*1*	LA+ Vocabulary Fluency				
*2*	LA+ Vocabulary FluencyLA- Vocabulary Fluency	0.002	>0.1	<0.001	>0.1
***Right IPS***					
*1*	LA+ Vocabulary Fluency				
*2*	LA+ Vocabulary FluencyLA- Vocabulary Fluency	0.040	>0.1	3.400	>0.1

For all regions of interest, language differences for performance on small problems and large problems were modeled separately. The additional variance explained, R^2^Δ, and corresponding*p*-values are shown.

#### 3.3.2 Effects of LA- vocabulary fluency: summary of results

In summary, we did not observe any significant relations between vocabulary fluency in LA- and language differences in arithmetic performance or brain activation. Our findings could reflect a lack of variability in the language skills of our balanced bilinguals.

#### 3.3.3 Bayesian modeling of language effects

For further confirmation that the languages did not differ within our regions of interest, we conducted analyses using Bayesian modeling. We modeled our brain data based on Problem Size, Language, and the additive effect with and without the interaction between the two. The Watanabe information criteria comparison (WAIC;Watanabe, 2013) was used to determine which model best fit the data, and posterior summaries of the model parameters provided Bayesian*p*-values, which reflect the likelihood of disconfirming a null effect (i.e., zero difference across conditions).*p*-Values were considered significant when the 95% highest posterior density for each parameter does not contain 0. To obtain the values that were input into the Bayesian models, we extracted the entire signal intensity for the contrast LA+ > control for small and large problems, separately, and LA- > control for small and large problems, separately, within our regions of interest as defined by our functional localizers (see theDefining Regions of Interest section). These values were extracted for each participant individually across the 4 conditions.

For all regions of interest, the WAIC comparison showed that the models without the interactions best fit the data. In brief, this analysis confirmed a main effect of problem size only within the STG/MTG. This effect was driven by greater temporal activation for small problems in relation to large problems. In contrast to our original planned analyses, this analysis also revealed a main effect of language within the voxels of the right IPS (seeTable 6). The effect in right IPS was driven by decreased activation for LA- than LA+.

**Table 6.** Bayesian*p*-values based on modeling contrast estimates from our regions of interest, left STG/MTG, left IFG, and bilateral IPS.

Region of interest	Main effect of language	Main effect of problem size	Interaction term
Left STG/MTG	56.27%	*100.00%	50.20%
Left IFG	85.70%	66.12%	50.10%
Left IPS	68.65%	88.48%	65.53%
Right IPS	*98.45%	75.80%	65.10%

Bayesian*p*-values reflect the likelihood of disconfirming a null effect (i.e., zero difference across conditions). *p-Values greater than 95% were considered significant.

Because the model for the right IPS suggested that there was a strong likelihood for disconfirming a null effect of Language, we extracted the contrast estimates only for the voxels that were significantly active for problem size for all of our regions of interest (with the exception of the left IPS, where no significant voxels were identified based on problem size). This analysis was done with small problems and large problems separately, comparing only LA+ and LA-. Modeling the data with these voxels did not change the pattern of results. For the right IPS, there was a Bayesian*p*-value of 99.65% for a main effect of Language for small problems and a Bayesian*p*-value of 96.12% for large problems, where LA- showed less activation than LA+ for both problem types.

#### 3.3.4 Bayesian modeling of language effects: summary of results

In summary, Bayesian modeling of our brain data suggested evidence of a main effect of Language within the voxels of the right IPS, where LA- showed decreased activation in comparison to LA+. Given that the right IPS is thought to be involved when quantity processes are engaged, these results were unexpected. However, one interpretation of our results is that balanced bilinguals automatically engage processes related to non-symbolic numerical magnitude to a lesser extent in their weaker language, LA-. We discuss this further in the discussion below.

#### 3.3.5 Analyses of only true problems (i.e., problems presented with correct solutions)

In our previous analysis of task performance (seeSection 3.1), we found a main effect of Language for response time that was driven by large problems, where bilinguals were faster responding to those problems in LA-. In addition to this finding, this analysis revealed a significant interaction between Correctness (i.e., true vs. false problems) with Problem size. Thus, to explore whether the original behavioral patterns were driven by problem correctness, we repeated our analyses of behavioral measures with only true multiplication problems.

For response time, the main effect of Language was no longer significant (*F*(1, 84) = 0.73;*p*> 0.1), but there was still a main effect of Problem size (*F*(1, 84) = 146.95;*p*< 0.001; η_p_^2^= 0.64). Additionally, the interaction between Language and Problem size was no longer significant (*F*(1, 84) = 1.77;*p*> 0.1). For accuracy, the main effect of Problem size remained significant (*F*(1, 84) = 45.75;*p*< 0.001; η_p_^2^= 0.35), where small problems were verified more accurately than large problems. The main effect of Language was not significant (*F*(1, 84) = 0.018;*p*> 0.1), and there was no interaction between Language and Problem size (*F*(1, 84) = 0.34;*p*> 0.1).

Given that including only true solutions revealed a different pattern of results for behavioral measures, we also repeated our hierarchical regression analyses exploring a relation between LA- fluency and language-related differences in arithmetic performance. As before, we completed the analysis with both large and small multiplication problems, separately. In these hierarchical regression analyses, we included LA+ vocabulary fluency in the model as a covariate of no interest and LA- vocabulary fluency as the independent variable. The dependent measure was the difference in task performance in multiplication (accuracy and response time, separately) across languages (i.e., LA+ accuracy – LA- accuracy or LA+ response time – LA- response time). For both response time and accuracy, there was no significant relation between language differences in performance and LA- vocabulary fluency, above and beyond the variance accounted for by LA+ vocabulary fluency.

We also repeated all of our previous analyses of brain activity, only including true solutions. The pattern of results did not change for either hypothesis 1 (main effect of Language) or hypothesis 2 (interaction between Language and Problem size), where no significant clusters were observed. Additionally, our hierarchical regression analyses did not reveal any significant relation between language differences in brain measures within our ROIs and LA- vocabulary fluency above and beyond LA+ vocabulary fluency, consistent with our previous analyses.

**Fig. 8.**
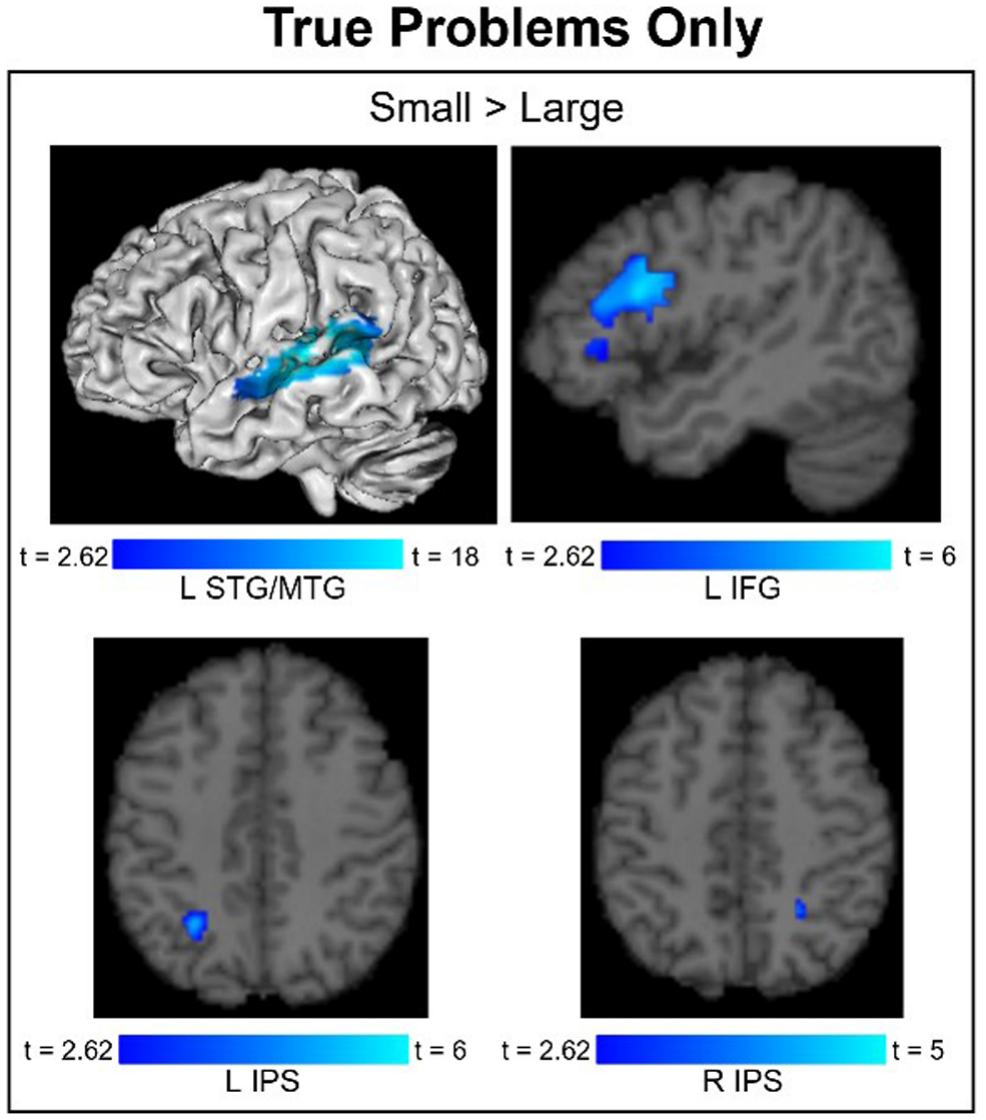
Main effects of problem size for true solutions within the regions of interest. Depiction of the brain activation for the contrast small > large for true solutions of the multiplication task, collapsed across languages. The significant cluster in left STG/MTG is shown on a rendered brain on the left hemisphere (top left). The significant clusters in the left IFG (top right), left IPS (bottom left), and right IPS (bottom right) are shown.

In contrast to our original results, the main effect of Problem size revealed significant clusters for the contrast small > large for all 4 regions of interest (Fig. 8), and no significant clusters were observed for the contrast large > small (SeeTable 7). The significant clusters in the left STG/MTG and left IFG were consistent with our original analyses; however, the clusters found in bilateral IPS were not.

**Table 7.** Cluster information for the main effect of problem size for the multiplication problems with true solutions within the regions of interest.

*Anatomical location*		MNI coordinates				
	*~BA*	*X*	*Y*	*Z*	*k*	*z* -value
Multiplication Task [small > large]						
Left STG/MTG	22/21	-62	-32	6	1,969	>8
Left IFG	44/45	-40	14	26	1,060	6.68
L IPS	7	-26	-56	44	113	4.76
R IPS	7	24	-52	48	23	3.54
Multiplication Task [large > small]						
No Significant Voxels						

Cluster size (k), MNI coordinates of the peaks, Z values, and approximate Brodmann areas (~BA) for the clusters showing significant activation for the problem size contrasts.

Similar to our previous results, modeling our data using Bayesian analyses confirmed a main effect of problem size within the STG/MTG and left IPS and revealed a main effect of Language within the voxels of the right IPS (seeTable 8).

**Table 8.** Bayesian*p*-values based on modeling contrast estimates from our regions of interest, left STG/MTG, left IFG, and bilateral IPS only including true trials.

Region of interest	Main effect of language	Main effect of problem size	Interaction term
Left STG/MTG	51.25%	*100.00%	65.18%
Left IFG	79.95%	85.75%	73.22%
Left IPS	64.62%	*96.58%	64.10%
Right IPS	*97.08%	90.80%	72.05%

Bayesian*p*-values reflect the likelihood of disconfirming a null effect (i.e., zero difference across conditions). *p-values greater than 95% were considered significant.

#### 3.3.6 Analyses of only true problems: summary of results

In summary, when we included only problems with correct (true) solutions in our analyses, we found that although the main effects of Problem size remained significant for measures of performance, the main effect of Language was no longer significant for response time. Additionally, the interaction between Problem size and Language for response time was no longer significant. For our measures of brain activation, there were still no significant clusters showing language differences in any of our regions of interest. Moreover, there were no relations between the size of language effects in performance or brain measures and LA- vocabulary fluency. Lastly, our Bayesian modeling results did not change after analyzing only true problems. These results suggest that the language differences we originally observed in performance may have been driven by problems with incorrect (false) solutions.

## 4 Discussion

The current study aimed to determine if adult Spanish-English bilinguals engage brain regions known to be involved in multiplication processing to the same extent in both of their languages. To address this overarching question, we measured the fMRI activation patterns in fluent Spanish-English bilingual adults as they verified the correctness of single-digit multiplication problems presented in the language that they learned arithmetic (LA+) or in their other language (LA-).

First, we hypothesized that LA+ would engage the left superior and middle temporal gyri (STG/MTG), associated with the verbal representation of math facts (Dehaene & Cohen, 1995;Prado et al., 2011), to a greater degree than LA-. We also predicted that LA- would recruit additional brain areas, including the left inferior frontal gyrus (IFG) reflecting more effortful retrieval (Prado et al., 2011) and/or the bilateral intraparietal sulcus (IPS) for reliance on quantity processing (Chassy & Grodd, 2012;Sokolowski et al., 2017).

Second, we examined the interaction between language of arithmetic learning and problem difficulty (i.e., problem size). Small multiplication problems (i.e., problems with solutions smaller than 25) are more likely to be verbally retrieved compared to large multiplication problems (i.e., problems with solutions larger than 25) (Siegler, 1987). Moreover, increased activity in temporal regions has been associated with increased expertise with arithmetic, particularly for more practiced, small problems (Prado et al., 2014). Thus, we hypothesized that an increase in STG/MTG for LA+ relative to LA- would be most prominent for small problems. In contrast, given that operating in a weaker language for arithmetic has been linked with the activation of a more extensive language network (Van Rinsveld et al., 2017), we hypothesized that LA- would recruit IFG to a greater extent than LA+, and that this would be most prominent when verifying larger (more difficult to retrieve) problems. Additionally, we predicted that LA- would recruit the IPS, reflecting reliance on quantity processes for a weaker arithmetic language. However, despite performance differences across languages, there were no differences in any of our brain measures based on language. We discuss this in further detail below.

### 4.1 Language bias for multiplication performance

As expected, verifying the correctness of multiplication problems with smaller solutions led to faster and more accurate responses than large problems, a typical problem size effect (seeZbrodoff & Logan, 2004for review). Additionally, our sample of early, proficient bilinguals was faster to verify problems presented in LA- compared to LA+. This pattern is inconsistent with many previous reports of a language bias in bilinguals (Cerda et al., 2019,^2023^;Dehaene et al., 1999;Frenck-Mestre & Vaid, 1993; Lotus Lin et al., 2019;Marsh & Maki, 1976;Salillas & Wicha, 2012;Spelke & Tsivkin, 2001;Tamamaki, 1993;Van Rinsveld et al., 2016,^2017^). These studies typically report that bilinguals are both faster, and more accurate at solving arithmetic problems in their LA+. Instead, we found evidence of slower responses to the language of learning arithmetic. The interaction between problem size and language was also significant for response time, where large problems in LA- were responded to more quickly than large problems in LA+. This suggests that the response time pattern across languages might be driven by the most difficult problems in our task. Critically, we did not find evidence that bilinguals perform differently on small problems across languages, likely because these problems are more frequently encountered overall, as they are typically taught first and appear more often in textbooks (Ashcraft & Christy, 1995).

In a follow-up exploratory analysis, we found evidence that the language differences in response time may have been driven by correctness judgments on problems with false solutions (e.g., 2 x 4 = 12), especially for larger problems. After reanalyzing our performance data, including only problems that were presented with true solutions (e.g., 2 x 4 = 8), there was no longer a significant main effect of Language or interaction between Language and Problem size for response time. Some have argued that the falsification of arithmetic problems involves separate cognitive processes from problem verification (Ashcraft & Battaglia, 1978;De Visscher et al., 2016). The false solutions in this study were always table-related to one of the two presented operands. Table-related false solutions are typically falsified more slowly than solutions that are unrelated to the operands, likely because associative priming from the proposed answer causes interference during the retrieval process (Romero et al., 2006). Thus, the inclusion of these types of solutions may have slowed processing in LA+ compared to LA- due to increased retrieval interference in LA+.

### 4.2 Language differences for arithmetic within regions of interest

For our functional imaging data, we hypothesized that we would observe differences in activation between LA+ and LA- within our regions of interest, including left STG/MTG, left IFG, and bilateral IPS. However, we did not find any evidence supporting these hypotheses from our planned analyses. Additionally, we did not find any evidence of an interaction between language and problem size within these regions.

Our findings seem to contradict recent neuroimaging evidence suggesting that bilinguals show additional activation for simple arithmetic processing in the language not used for arithmetic learning compared to their language of learning (Van Rinsveld et al., 2017;Venkatraman et al., 2006;Wang et al., 2007). For example,Van Rinsveld et al. (2017)used a whole-brain approach to investigate language differences for simple addition (i.e., with operands 2-8) and more complex addition (i.e., with operands 12-86) presented as auditory number words. The participants in their sample were German-French bilinguals who received all their early instruction, including early simple arithmetic, in German (LA+). For simple additions, the authors observed greater activation of temporal regions in German than French, suggesting that there were increased semantic associations of problems and solutions in German (LA+), but not French (LA-). For complex problems, the authors observed a broader network of activation in French than German, particularly in occipital-temporal regions, suggesting that French activated the visual representations of the numbers to solve complex problems.

Our neuroimaging results of no evidence for language differences in multiplication processing are in line with recent work suggesting that language background factors play a critical role in the size of language learning effects for arithmetic (Garcia et al., 2021;Tamamaki, 1993). Lower proficiency in a second language typically leads to larger differences in performance across languages on arithmetic tasks, whereas bilinguals with higher second language proficiency tend not to show performance differences across languages (seeCerda & Wicha, 2023, for review; see alsoGarcia et al., 2021). Moreover, in a recent meta-analysis,Garcia et al. (2021)suggested that when the first-learned language is also the language of learning for math (i.e., L1 = LA+), the effects of language on arithmetic performance tend to be more pronounced. The influence of language background factors may explain the language effects observed by previous neuroimaging studies investigating bilingual arithmetic. Indeed, the participants in theVan Rinsveld et al. (2017)study described above began learning French in secondary school and appeared to become proficient in French by the time they entered college. As a result, they learned simple arithmetic before becoming proficient in their two languages, likely driving more efficient processing in German as their native language and language of learning arithmetic. Similarly,Wang et al. (2007)tested native speakers of Chinese who learned English through course instruction at 12 years old on average, after the typical age of learning simple arithmetic. They found that the second language involved additional neural activation, especially in language areas, when performing addition and multiplication. These two findings support the suggestion ofGarcia et al. (2021)of more pronounced language effects in arithmetic performance when the language of math learning is also the first learned language.

Thus, it is possible that we did not observe significant language effects due to the balanced language abilities of our early, bilingual participants. Recently,Cerda et al. (2022)measured event-related potentials (ERPs) from fluent, early Spanish-English bilingual adults, using a very similar experimental task as the one used herein. They observed ERP modulations that were equivalent in timing, distribution, and size in both LA+ and LA-, suggesting that balanced adult bilinguals can categorize problems in both languages using the same cognitive processes, with equivalent efficiency. Our analysis of the effect of LA- language proficiency on arithmetic processing revealed no significant relations between LA- vocabulary fluency and performance on our math task or brain data during math. However, this could reflect the lack of variability in both LA+ and LA- language skills of our balanced bilingual adults.

It is also important to note that all participants identified English as their preferred language to do arithmetic in adulthood, even if they had originally learned math in Spanish (except for 2 individuals who reported context-dependent language preferences). Previous work has suggested that increased experience with arithmetic in a language that was not used for arithmetic learning might also mitigate differences across languages (seeMartinez-Lincoln et al., 2015). Thus, it is possible that the subgroup of participants who learned arithmetic in English (i.e., language arithmetic learning in childhood is the preferred language of arithmetic in adulthood) would show more robust language differences than the subgroup who learned arithmetic in Spanish (i.e., the language of arithmetic learning is not the preferred language in adulthood). However, our data suggest that balanced bilinguals on average do not show robust differences in the brain regions they engage for arithmetic across languages. Future work is needed to tease apart potential interactions between the language of arithmetic learning in childhood and preference for arithmetic in adulthood.

Although recent work suggests that balanced bilinguals are less likely to show robust language differences for arithmetic, there is electrophysiological evidence that language differences can arise under limited circumstances, such as when bilinguals are asked to verbally produce solutions across languages or when processing large problems (Cerda et al., 2023). Given this evidence, we might have expected that our balanced bilinguals would show a significant interaction between language and problem size. However, we did not observe language differences within the regions of interest, nor at the whole-brain level in an exploratory analysis.

To confirm that the languages did not differ within our regions of interest, we performed an exploratory analysis using Bayesian modeling on our brain data. In contrast to the results of our planned analyses, we found evidence of a main effect of language within the voxels of the right IPS, where LA- showed decreased activation relative to LA+. The effect persisted even after removing false problems from the analysis and appeared for both small and large problems. This pattern was unexpected, given that the right IPS is thought to be involved in the representation of non-symbolic numerical magnitude (Ansari, 2016;Faye et al., 2019) and has previously been implicated in processes related to calculation (Arsalidou & Taylor, 2011;Ashkenazi et al., 2012;Kucian et al., 2008) and quantity manipulation (Chassy & Grodd, 2012;Sokolowski et al., 2017). Because bilinguals learn and rehearse their multiplication facts primarily (or only) in LA+, it was predicted that they would rely more heavily on verbal retrieval in that language and less on quantity-based procedures.

It is important to note that the functional localizer we used to identify IPS was a dot comparison task. This is a task that is frequently used to measure non-symbolic magnitude processing and does not directly measure calculation or quantity manipulation. Thus, one interpretation of our Bayesian modeling results is that balanced bilinguals automatically engage processes related to non-symbolic numerical magnitude to a lesser extent in their weaker arithmetic language, LA-, than LA+. Generally, it is thought that number representations become less associated with their non-symbolic or quantity representations as individuals become more experienced with arithmetic (Bulthé et al., 2018). However, it is possible that adult bilinguals have developed automaticity for using quantity-based strategies in their LA-, since these problems are more difficult to retrieve. Recent work has shown that decreases in parietal cortex activity are associated with improvement in performance on subtraction problems, taken as support of quantity manipulation processes becoming more automatic/efficient with gains in skill (Suárez-Pellicioni et al., 2020). Future work is necessary to determine the dynamics of this effect, especially given that we did not observe this pattern within IPS in our original planned analyses.

### 4.3 Main effect of problem size

Because we did not find a significant interaction between language and problem size, we completed an additional exploratory analysis to directly measure the effect of problem size collapsing across languages. This analysis revealed greater activation for small problems than large problems in verbal areas (left STG/MTG and left IFG) and greater activation for large problems than small problems for quantity regions (right IPS).

The problem size effects within the left STG/MTG and right IPS are consistent with previous work. Temporal regions, including left STG/MTG, have been thought to be heavily involved in the storage of arithmetic facts in verbal memory, especially when they have been explicitly memorized (Prado et al., 2013). Moreover, increased activity in temporal regions is associated with increased expertise in arithmetic facts, particularly for smaller, more practiced multiplication problems (Prado et al., 2014). This pattern has been interpreted as a greater strengthening of semantic associations between problems and their solutions. Conversely, evidence suggests that the engagement of the right IPS increases with arithmetic task difficulty where problems with an increasing number of operands engage IPS to a greater extent (Menon et al., 2000). Additionally, problems that are more likely to involve indirect strategies during processing (i.e., decomposition, calculation, or transformation) tend to engage IPS to a greater extent than problems that rely only on retrieval (Prado et al., 2013). As discussed above, the region of the right IPS that we localized for analysis is likely involved in the representation of non-symbolic numerical magnitude. Although we cannot conclusively determine whether bilinguals are engaging in other backup strategies like calculation for larger problems, they seem to be more reliant on processes related to numerical magnitude as compared to small problems.

In contrast to the expected problem size effects in temporal-parietal regions, we did not expect small problems to engage the left IFG to a greater extent than large problems. Typically, left IFG has been associated with processes related to effortful retrieval of arithmetic facts, especially for facts that do not have robust memory representations in temporal cortices (Prado et al., 2011). Moreover,Prado et al. (2014)found that as children master multiplication facts, there are decreases in activity in the left IFG. The authors postulated that decreased engagement of left IFG might reflect a decrease in the reliance on executive control when processing arithmetic. In contrast, our data suggest that although bilinguals rely on retrieval of small multiplication facts in memory, indicated by greater engagement of temporal regions, they are also relying on more effortful retrieval processes. It is possible that having to manage two systems of multiplication fact representations is in itself more cognitively demanding than having only one (i.e., monolinguals), which could be associated with having to inhibit one of the languages while the other is being used. Critically, this does not seem to be dependent on the language the problems are presented in (as discussed above). It is possible that the left IFG is not engaged as much for large problems because our participants were more reliant on processes related to non-symbolic numerical magnitude than small problems, evidenced by the activation within IPS.

As mentioned, the problem size effect pattern within the left IFG was unexpected in our bilingual sample given previous work in monolingual adults and children. However, it is unclear whether these differences are due to the language background of our sample or differences in our experimental design that may have driven different processing. Previous fMRI studies in monolingual adults and children typically present multiplication problems as all Arabic digits (i.e., 2 x 3 = 6) (Berteletti et al., 2014;Demir-Lira et al., 2020;Prado et al., 2011,^2013^,^2014^;Suárez-Pellicioni et al., 2021;Suárez‐Pellicioni & Booth, 2022). The increased demand for hearing the problems might have contributed to the reliance on more effortful retrieval even for small multiplication problems and promote increased reliance on quantity processes for large problems.Van Rinsveld et al. (2017)presented bilinguals with simple and more complex addition problems as auditory number words in two languages. They also found left IFG activation, specifically in the the pars opercularis (BA 44; x = −42, y = 7, z = 7) for simple additions. Moreover, previous ERP studies have suggested that processing multiplication problems as Arabic Digits engages different or more efficient processing than cross-modal auditory multiplication tasks in monolinguals (Dickson et al., 2018) and bilinguals (Cerda et al., 2022).

## 5 Conclusions

Through a combination of planned and exploratory analyses, we did not find strong evidence for language differences within verbal and quantity areas known to be involved in arithmetic processing. Further, we did not find an interaction between the language of learning arithmetic and problem size. This suggests that balanced bilingual adults engage similar brain regions across languages, even for more difficult problems. We did find a main effect of problem size, where small problems recruited left STG/MTG and left IFG to a greater extent than large problems, suggesting greater verbal involvement for these problems. Conversely, large problems recruited the right IPS to a greater extent than small problems, suggesting a greater reliance on processes related to non-symbolic numerical magnitude for these problems. Overall, our results may be reflective of our bilingual’s balanced language abilities, where individuals with less balanced abilities or who learned their second language later in life may be more likely to show language differences. These findings highlight that it is critical to consider factors of a bilingual’s language background in understanding how the brain processes foundational concepts that are learned in the early years of schooling.

## Data Availability

All analyses were preregistered on Open Science Framework (OSF) (seehttps://osf.io/vbtr3). Additionally, all data and code used are shared on the associated project folder on OSF (osf.io/z9ajt).
